# Efficient Graph Collaborative Filtering via Contrastive Learning

**DOI:** 10.3390/s21144666

**Published:** 2021-07-07

**Authors:** Zhiqiang Pan, Honghui Chen

**Affiliations:** Science and Technology on Information Systems Engineering Laboratory, National University of Defense Technology, Changsha 410073, China; panzhiqiang@nudt.edu.cn

**Keywords:** recommender systems, efficient recommendation, collaborative filtering, graph convolution networks, contrastive learning

## Abstract

Collaborative filtering (CF) aims to make recommendations for users by detecting user’s preference from the historical user–item interactions. Existing graph neural networks (GNN) based methods achieve satisfactory performance by exploiting the high-order connectivity between users and items, however they suffer from the poor training efficiency problem and easily introduce bias for information propagation. Moreover, the widely applied Bayesian personalized ranking (BPR) loss is insufficient to provide supervision signals for training due to the extremely sparse observed interactions. To deal with the above issues, we propose the Efficient Graph Collaborative Filtering (EGCF) method. Specifically, EGCF adopts merely one-layer graph convolution to model the collaborative signal for users and items from the first-order neighbors in the user–item interactions. Moreover, we introduce contrastive learning to enhance the representation learning of users and items by deriving the self-supervisions, which is jointly trained with the supervised learning. Extensive experiments are conducted on two benchmark datasets, i.e., Yelp2018 and Amazon-book, and the experimental results demonstrate that EGCF can achieve the state-of-the-art performance in terms of Recall and normalized discounted cumulative gain (NDCG), especially on ranking the target items at right positions. In addition, EGCF shows obvious advantages in the training efficiency compared with the competitive baselines, making it practicable for potential applications.

## 1. Introduction

Recommender systems can help provide users with personalized information from the explosively increasing information on the internet [1,2,3], which have been widely applied in web search, e-commerce websites, etc. [4,5]. The core of a personalized recommender is to accurately capture the user preference from her historical interactions with items [2,6]. As an effective solution, collaborative filtering (CF) aims to learn accurate representation of users and items by reconstructing the user–item interaction matrix, then item recommendations can be generated by ranking the items according to the learnt user and item latent factors [1,7].

Existing methods generally generate the representation of users and items from two aspects, i.e., the self information represented by the ID embeddings and the collaborative information propagated through the user–item interactions, respectively. For example, matrix factorization (MF) projects users and items into a shared latent space and utilizes the inner product to make predictions [8]. Moreover, He et al. extend MF by utilizing the non-linear functions to learn the user–item interaction function [1]. In addition, considering user’s historical interactions can reflect her interest, factored item similarity models (FISM) [9] generates the user representation by combining the embeddings of the interacted items as user’s collaborative information in a weighted way. Furthermore, recently graph neural networks (GNN) are introduced into collaborative filtering by transforming user’s interactions with items into a user–item bipartite graph. For example, Wang et al. propose neural graph collaborative filtering (NGCF) which adopts graph convolution network (GCN) [10] to model the high-order connectivity between users and items by propagating collaborative information for both users and items on the bipartite graph [2]. Then, He et al. design LightGCN, which simplifies NGCF by merely preserving the neighborhood aggregation in GCN to improve its applicability for recommendation, achieving the state-of-the-art performance [11].

Though the aforementioned methods have achieved considerable performance, there still remains several limitations. First, the state-of-the-art GNN-based methods for collaborative filtering adopt multi-layer GNNs to propagate information from high-order neighbors in the user–item bipartite graph, which has a low computational efficiency. Moreover, unrelated neighbors including both users and items are easily introduced, which brings much bias, disturbing the detection of user interest. In addition, the widely adopted Bayesian personalized ranking (BPR) loss can merely adopt the supervision signals from the user–item interactions for training, which is insufficient due to the data sparsity problem [12].

To solve the above issues, we propose the Efficient Graph Collaborative Filtering (EGCF) method. Specifically, given historical user–item interactions, we first construct a user–item bipartite graph. Then on the one hand, we propagate embeddings from the first-order neighbors of users and items to obtain the collaborative information, which are combined with the self information (i.e., the ID embeddings of users and items), to generate their corresponding representations. On the other hand, we introduce contrastive learning by adopting information noise-contrastive estimation (InfoNCE) [13] to derive the self-supervisions between users and between items, so as to simultaneously enhance the representation learning of users and items and establish the high-order connectivity in the bipartite graph.

Extensive experiments are conducted on two publicly available datasets, i.e., Yelp2018 and Amazon-book. The experimental results demonstrate that EGCF can achieve significant improvements above the state-of-the-art baselines in terms of Recall and NDCG.

The main contributions in this paper are summarized as follows:

1. We propose an Efficient Graph Collaborative Filtering (EGCF) method, which simplifies the GNN-based CF methods by preserving merely one-layer graph convolution to propagate collaborative information for improving the computational efficiency;
2. We introduce constrastive learning into graph collaborative filtering to enhance the representation learning of users and items and take the high-order connectivity between users and items into consideration;
3. Comprehensive experiments conducted on two benchmark datasets, i.e., Yelp2018 and Amazon-book, demonstrate that EGCF can achieve the state-of-the-art performance in terms of Recall and NDCG.

We organize the rest of this paper as follows: We summarize the related literature in Section 2. Then in Section 3, we describe our proposed EGCF method in detail. After that, we give the experimental settings in Section 4 and conduct deep analysis of the experimental results in Section 5. Finally, we conclude our work and suggest several future directions in Section 6.

## 2. Related Work

In this section, we first summarize the previous work about the collaborative filtering in Section 2.1, then we review the related work about constrastive learning and its applications in recommender systems in Section 2.2.

### 2.1. Collaborative Filtering

Previous work for collaborative filtering can be mainly divided into three categories, i.e., the MF-based methods, the history-aware methods, and the GNN-based methods. As for the MF-based methods, the common paradigm is to embed users and items into low-dimensional latent vectors, and then learn the embeddings by reconstructing the historical user–item interactions [8,14]. Moreover, recently neural networks are introduced into MF for enhancing the user–item interaction modeling [1,15]. Upon merely utilizing the ID embeddings, the history-aware methods are proposed to include user’s historical interacted items for detecting the user intent [9,16,17,18]. For example, FISM [9] combines the embeddings of interacted items as user’s preference, then attentive collaborative filtering (ACF) [17] and neural attentive item similarity (NAIS) model [18] further are proposed to automatically determine the importance of each historical item in an attentive way. Recently, graph neural network (GNN) [10,19,20] are introduced into CF. For instance, He et al. propose NGCF which adopts the GCN to model the collaborative signal by exploiting the high-order connectivity between users and items [2]. Moreover, Wang et al. propose to introduce the disentangled learning into CF to consider the finer granularity of user intents on items [21]. In addition, He et al. simplify NGCF by preserving merely the neighborhood aggregation in GCN to make it more appropriate for recommendation [11].

However, the MF-based and history-aware methods are insufficient to model the collaborative signal effectively, and the GNN-based methods suffer from the low efficiency problem and easily introduce bias, making them fail to simultaneously achieve high accuracy and efficiency for recommendation.

### 2.2. Contrastive Learning

Contrastive learning aims to learn high-quality representations by comparing the local and global representations of a training sample or contrasting the representations of the same sample from different views, which has been widely applied in computer vision [22,23,24] and natural language processing [25,26], etc. Considering the effectiveness of contrastive learning in various fileds, recently constrastive learning has also introduced into recommender systems for learning the accurate representation of users and items. For instance, Zhou et al. propose to maximize the mutual information between different forms or granularities of the item sequence to enhance the item representation learning for improving the sequential recommendation task [27]. Moreover, Xie et al. propose to enhance the supervised learning with constrastive learning in a pre-training way, which extracts the self-supervision signals by contrasting the same item sequence from various views generated by different augmentation methods [28]. In addition, Ma et al. propose to perform the self-supervisions in the disentangled latent space by a sequence-to-sequence training strategy to simultaneously extract additional supervision signals and exploit user’s multi intents [29].

However, limited work has been conducted to derive additional self-supervisions for the user and item representation learning on the collaborative filtering task. Thus, in this paper, we introduce constrastive learning into CF to simultaneously improve the recommendation accuracy and the training efficiency.

## 3. Approach

In this section, we first formulate the collaborative filtering task. Then, we detail our proposed Efficient Graph Collaborative Filtering (EGCF), which mainly consists of three components, i.e.,  the graph convolution module, the supervised and contrastive learning module, and the joint learning module.

**Figure 1:**
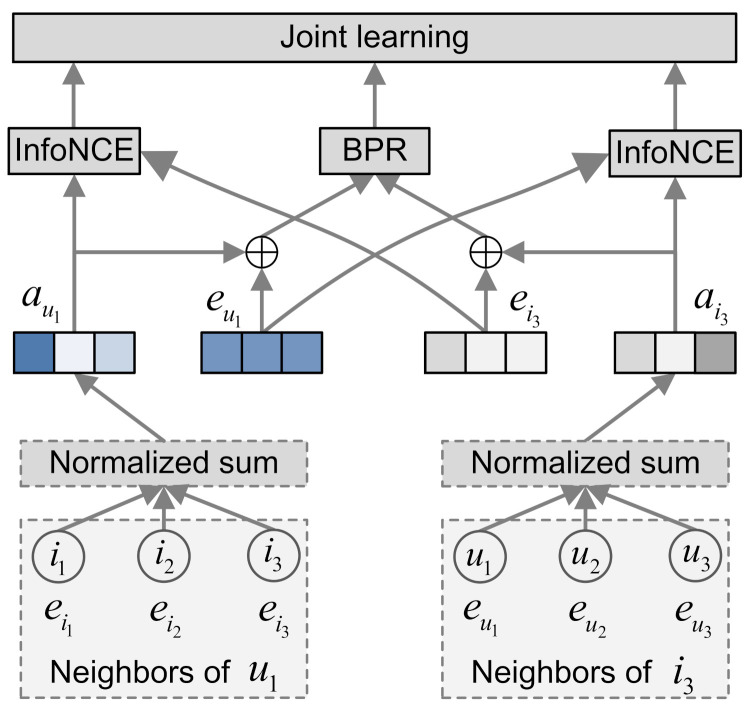
The framework of EGCF.

The framework of our proposed EGCF is plotted in Figure 1. Given the historical user–item interactions, we first construct a bipartite graph according to the interaction relation. Then, for each node (including users and items), we propagate information from the first-order neighbors using the simplified graph convolution networks (SGCN), which is then combined with their self information (i.e., the ID embeddings) to generate the final representation of users and items. After that, for each user–item pair, on the one hand, we adopt the Bayesian personalized ranking (BPR) loss as the main supervised loss, aiming to reconstruct the observed interactions; on the other hand, we derive the self-supervisions between users and between items to enhance the user and item representations by the InfoNCE. Finally, we perform joint learning by combining both the supervised and contrastive losses.

Assuming U and I are the user set and item set, respectively. Let $O^{+}$ be the interaction set between U and I, where $y_{ui}\in O^{+}$ indicates that user u∈U interacted with item i∈I. The aim of collaborative filtering is to learn user and item representations from the interaction set $O^{+}$, so as to predict the likelihood of each user adopting the candidate items in I, then items ranking at the top positions will constitute the recommendation list for the user.

We list the main notations used in this paper in Table 1.

### 3.1. Graph Convolution

Given the interaction set $O^{+}$ between users U and items I, we first construct a user–item bipartite graph. Let G={V,E} denotes the constructed graph, where V indicates the node set including all users and items, and $E=O^{+}$ denotes the observed interactions between users and items. Specifically, each edge $(e_{u},e_{i})\in E$ indicates that user *u* interacted with item *i* before.

After constructing the user–item bipartite graph, we propagate information from the first-order neighbors of each node to update the node vector using one-layer graph convolution. Specifically, we perform the information propagation using the simplified graph convolution networks (SGCN) proposed in [11], which merely preserves the neighborhood aggregation in GCN and shows competitive performance in recommendation. Specifically, we first propagate information from the neighbors of each user and item. The graph convolution operation for each user and item can be formalized as follows:

(1) $$\begin{array}{cc} a_{u} & =\sum_{i\in N_{u}}\frac{1}{\sqrt{|N_{u}|}\sqrt{|N_{i}|}}e_{i},\\ a_{i} & =\sum_{u\in N_{i}}\frac{1}{\sqrt{|N_{i}|}\sqrt{|N_{u}|}}e_{u}, \end{array}$$

where $a_{u}\in R^{d}$ and $a_{i}\in R^{d}$ are the propagated information for user *u* and item *i*, respectively, and $e_{u}\in R^{d}$ and $e_{i}\in R^{d}$ are their corresponding ID embeddings. $N_{u}$ and $N_{i}$ are the neighbors of user *u* and item *i*, respectively, where the neighbors are the interacted items for each user and the users who interacted with for each item. $\frac{1}{\sqrt{|N_{u}|}\sqrt{|N_{i}|}}$ and $\frac{1}{\sqrt{|N_{i}|}\sqrt{|N_{u}|}}$ are the symmetric normalization terms for avoiding the scale of embeddings increasing with the graph convolution operation.

### 3.2. Supervised and Contrastive Learning

#### 3.2.1. Supervised Learning

After propagating information from the neighbors of each node in the user–item bipartite graph, we combine the propagated information with the self information to generate the final node representation as follows:

(2) $$\begin{array}{cc} z_{u} & =e_{u}+a_{u},\\ z_{i} & =e_{i}+a_{i}, \end{array}$$

where $z_{u}\in R^{d}$ and $z_{i}\in R^{d}$ are the generated final representation of user *u* and item *i*, respectively. Here we adopt the sum pooling following [11] considering its simplicity since it can avoid introducing additional learnable parameters, and its effectiveness has also been widely validated in multi work [11,12].

After generating the representation of users and items, we then make predictions by a inner product following [2,11,21] as follows:

(3) $${\hat{y}}_{ui}={z_{u}}^{T}z_{i},$$

where ${\hat{y}}_{ui}$ is the predicted score of measuring how likely user *u* would adopt item *i*.

Then, to learn the trainable parameters in EGCF (i.e., the ID embeddings of users and items), we employ the pairwise Bayesian personalized ranking (BPR) loss as the optimization objective in the supervised learning. Specifically, the BPR loss adopts the observed interactions as the supervision signals, encouraging the predicted score of each interaction, i.e., ${\hat{y}}_{ui}$ to be close to the ground truth, i.e., $y_{ui}$. The optimization objective can be formalized as follows:

(4) $$L_{s}=\sum_{(u,i,j)\in O}-\log\left(\sigma \left({\hat{y}}_{ui}-{\hat{y}}_{uj}\right)\right),$$

where $L_{s}$ indicates the supervised loss, ${\hat{y}}_{ui}\in O^{+}$ is the observed interaction while ${\hat{y}}_{uj}\in U\times I\backslash O^{+}$ is the randomly sampled unobserved interaction, and σ denotes the sigmoid function. By enlarging the distance between the prediction score of the observed and unobserved interactions, the model can push user’s interacted items at an earlier position.

#### 3.2.2. Contrastive Learning

Through propagating information from the one-hop neighbors in the user–item bipartite graph, we can incorporate the collaborative information for representing the users and items. Then, in order to learn better user and item representations and take the high-order connectivity between users and items into consideration, we introduce constrastive learning to derive the self-supervision signals for the model optimization.

Specifically, for each user–item interaction $y_{ui}$, we can obtain the self and the neighbor information as $e_{u}$ and $a_{u}$ for each user, and the corresponding representations for each item are $e_{i}$ and $a_{i}$, respectively. For each user, the ID embddings of user *u*’s interacted item *i* in $y_{ui}$ (i.e., $e_{i}$) is part of the neighbor information of user *u* (i.e., $a_{u}$ which fuses the embeddings of the interacted items $N_{u}$), since $i\in N_{u}$. Then, we introduce constrastive loss based on the intuition that item *i* is more similar to user *u*’s neighbors than other users’ neighbors. Specifically, considering the current mini-batch is consisted of *N* user–item interactions as $\left[\left(u_{1},i_{1}\right),\left(u_{2},i_{2}\right),\ldots ,\left(u_{N},i_{N}\right)\right]$, then for user $u_{\epsilon }$, we introduce the InfoNCE [13] as the contrastive loss, which treats $\left(i_{\epsilon },N_{u_{\epsilon }}\right)$ as the positive pair and regards the pairs combining item $i_{\epsilon }$ with the neighbors of other users in the mini-batch (i.e., $\left[\left(i_{\epsilon },N_{u_{\kappa }}\right)|\kappa =1,\ldots ,\epsilon -1,\epsilon +1,\ldots ,N\right]$) as the negative samples:

(5) $$L_{c}^{i}=\frac{\exp(\lambda \mathrm{sim}\left(e_{i_{\epsilon }},a_{u_{\epsilon }}\right))}{\exp\left(\lambda \mathrm{sim}\left(e_{i_{\epsilon }},a_{u_{\epsilon }}\right)\right)+\sum_{\kappa =1,\kappa \neq \epsilon }^{N}\exp\left(\lambda \mathrm{sim}\left(e_{i_{\epsilon }},a_{u_{\kappa }}\right)\right)},$$

where $L_{c}^{i}$ is the contrastive loss from the item side. $\mathrm{sim}\left(u,v\right)=cos\left(u,v\right)=u^{T}v/\left||u||||v|\right|$ is the similarity measuring function, ||·|| denotes the L2 normalization, and λ is a hyper-parameter for scaling the cosine similarity.

Similarly, for item $i_{\epsilon }$ in the current mini-batch, we treat $\left(u_{\epsilon },N_{i_{\epsilon }}\right)$ as the positive pair and regard the pairs combining user $u_{\epsilon }$ with the neighbors of other items in the mini-batch (i.e., $\left[\left(u_{\epsilon },N_{i_{\kappa }}\right)|\kappa =1,\ldots ,\epsilon -1,\epsilon +1,\ldots ,N\right]$) as the negative samples. Then the contrastive loss from the user side can be defined as:

(6) $$L_{c}^{u}=\frac{\exp(\lambda \mathrm{sim}\left(e_{u_{\epsilon }},a_{i_{\epsilon }}\right))}{\exp\left(\lambda \mathrm{sim}\left(e_{u_{\epsilon }},a_{i_{\epsilon }}\right)\right)+\sum_{\kappa =1,\kappa \neq \epsilon }^{N}\exp\left(\lambda \mathrm{sim}\left(e_{u_{\epsilon }},a_{i_{\kappa }}\right)\right)},$$

where $L_{c}^{u}$ is the contrastive loss from the user side.

Finally, we combine both the item- and user-side contrastive losses as the final contrastive loss $L_{c}$ as follows:

(7) $$L_{c}=L_{c}^{i}+L_{c}^{u},$$

Through the item-side contrastive leaning, we can encourage items interacted by the same user generate similar representations, such as item $i_{3}$ and $i_{1}$ in Figure 1. Similarly, the user-side self-supervisions enforce users who interacted with the same item to be similar in the latent space, like user $u_{1}$ and $u_{2}$ in Figure 1. By combining both the item-side and user-side contrastive leaning, we can take the high-order connectivity between users and items into consideration by learning smoothing representations for similar users and similar items. Different from the previous work relying on multi-layer GNNs to propagate high-order embeddings, our proposal merely adopts one-layer graph convolution, which can largely improve the training efficiency. We will detail the computational complexity analysis theoretically in Section 3.4 and evaluate the training efficiency of EGCF empirically in Section 5.2, respectively.

### 3.3. Joint Learning

After obtaining the supervised loss $L_{s}$ in Equation (4) and the contrastive loss $L_{c}$ in Equation (7), we perform joint learning by combining them as follows:

(8) $$L=L_{s}+\alpha L_{c},$$

where α is a hyper-parameter for controlling the weight between the supervised and contrastive losses. Finally, we adopt the Back-Propagation Through Time (BPTT) algorithm [30] to optimize the final loss L for learning the trainable parameters in EGCF.

To illustrate the training process of EGCF, we detail the learning algorithm of EGCF in Algorithm 1. Given the observed interactions $O^{+}$ between users U and items I, we first construct the user–item bipartite graph in line 1. Then for each user–item pair in the interactions of the current minibatch (i.e., $O_{B}^{+}$), we sample the negative item in line 5, and then initialize the embeddings of user *u* as well as items *i* and *j* in line 6. After that, we perform graph convolution to obtain the neighbor information of user and items in line 7, which is combined with the ID embeddings to generate their final representations in line 8. Next, we optimize the model using two types of supervisions: (1) On the one hand, we make predictions in line 9 and adopt the BPR loss as the supervised loss in line 10; and (2) on the other hand, we introduce the InfoNCE to derive the self-supervisions from item- and user-side in line 11 and 12, respectively, which are then combined as the final contrastive loss in line 13. Finally, we generate the joint training loss in line 15 and adopt the back-propagation to learn the model parameters in line 16.
**Algorithm 1.** Learning algorithm of EGCF.Input:The observed interactions between users and items, i.e., $O^{+}$;Output:The embeddings of users and items in EGCF;1:G={V,E}←GraphConstruct ($O^{+}$);2:**for** epoch in 1, …, s **do**3:    **for** minibatch M∈X **do**4:        **for** (u,i) in $O_{M}^{+}$ **do**5:             j←RandomSample(u);6:             $e_{u},e_{i},e_{j}\leftarrow \mathrm{EmbeddingLayer}\left(u,i,j\right)$;7:             $a_{u},a_{i},a_{j}\leftarrow \mathrm{SGCN}\left(N_{u},N_{i},N_{j}\right)$ based on Equation (1);8:             $z_{u},z_{i},z_{j}\leftarrow \mathrm{Combination}\left(\left[e_{u},a_{u}\right],\left[e_{i},a_{i}\right],\left[e_{j},a_{j}\right]\right)$ based on Equation (2);9:             ${\hat{y}}_{ui},{\hat{y}}_{uj}=\mathrm{Prediction}\left(z_{u},z_{i},z_{j}\right)$ based on Equation (3);10:           $L_{s}=\mathrm{BPR}\left({\hat{y}}_{ui},{\hat{y}}_{uj}\right)$ based on Equation (4);11:           $L_{c}^{i}=\mathrm{InfoNCE}\left(e_{i},a_{u},a_{k}\right)$, $k\in U_{M}\backslash u$ based on Equation (5);12:           $L_{c}^{u}=\mathrm{InfoNCE}\left(e_{u},a_{i},a_{k}\right)$, $k\in I_{M}\backslash i$ based on Equation (6);13:           $L_{c}=L_{c}^{i}+L_{c}^{u}$ based on Equation (7);14:        **end for**15:        Optimize joint learning loss: $L=L_{s}+\alpha L_{c}$;16:        use back-propagation to optimize the parameters;17:    **end for**18:**end for**19:**return** User and item embeddings E.

### 3.4. Model Complexity Analysis

To prove the efficiency of EGCF theoretically, we analyze the computational complexity of our proposal as well as the state-of-the-art baseline LightGCN. The results are provided in Table 2, where we compute the time complexity of different components in two models. The complexity consists of mainly four parts:

- Adjacency matrix normalization. After constructing the adjacent matrix of the user–item bipartite graph, the weights need to be normalized, which consumes a complexity of 2|E|, where |E| is the interaction number;
- Graph convolution. Let *s* and *B* denote the number of epochs and the size of each training mini-batch, then the complexity of the graph convolution is $O(2|E|Lds\frac{\left|E\right|}{B})$ for LightGCN while $O(2|E|ds\frac{\left|E\right|}{B})$ for EGCF since we reduce the layer number of graph convolution to 1;
- Supervised loss. As for the supervised loss produced by Equation (4), LightGCN and EGCF share the same complexity, i.e., O(2|E|ds);
- Contrastive loss. Compared with LightGCN, the additional complexity of contrastive loss in EGCF can be denoted as O((2+2B)|E|ds), which comes from the item side O((1+B)|E|ds) and the user side O((1+B)|E|ds), respectively.

As we can observe, comparing to LightGCN, EGCF reduces the complexity of graph convolution by $O(\left(L-1\right)\frac{\left|E\right|}{B}\cdot 2|E|ds)$ and adds an additional complexity O((1+B)·2|E|ds) from constrastive learning. Since the interaction number |E| can reach several millions as shown in Table 3, and the batch size *B* is usually set to several thousands like 1024, 2048, etc., thus the two complexities $O(\left(L-1\right)\frac{\left|E\right|}{B}\cdot 2|E|ds)$ and O((1+B)·2|E|ds) are similar numerically.

In addition, during training, most of the consuming time comes from the back propagation for updating the parameters [30]. For the previous GNN-based methods, taking the high-order neighbors will make the embeddings of the introduced neighbors also be updated in the back propagation. Differently for EGCF, we reduce the layer number of graph convolution to 1, and the self-supervision signals in constrastive learning are also produced from the current mini-batch, thus can effectively avoid the above-mentioned problem and obviously reduce the training time. We will prove the training efficiency of EGCF empirically in Section 5.2.

## 4. Experiments

### 4.1. Research Questions

We prove the effectiveness and efficiency of EGCF by addressing the following four research questions:

(RQ1) Can our proposed EGCF outperform the competitive baselines on the collaborative filtering task?
(RQ2) How is the training efficiency of EGCF compared with the state-of-the-art baseline LightGCN?
(RQ3) How does each component in EGCF contribute to the recommendation accuracy of EGCF?
(RQ4) What is the impact of the trade-off parameters including α and λ on the performance of EGCF?

### 4.2. Datasets and Evaluation Metrics

We adopt two publicly available datasets, i.e., Yelp2018 and Amazon-book, to evaluate the performance of EGCF and the baselines. Yelp2018 comes from the Yelp challenge in 2018, which collects the interactions between users and the local businesses such as restaurants and bars, here the businesses are regarded as items following [2,11]. Amazon-review is a dataset widely used for product recommendation, here Amazon-book is selected for our experiments as in [2,21]. Moreover, for both Yelp2018 and Amazon-book, we adopt the 10-score setting [31] to ensure the data quality, which filters users with less than 10 interactions and items appearing less than 10 times. In addition, following [2,11], we randomly select 80% of the historical interactions as the training set, and the remaining part is adopted as the test set. Furthermore, from the training set, we randomly separate 10% of interactions as the validation set for tuning the hyper-parameters. Finally, 31,668 users and 38,048 items as well as 1,561,406 interactions are remained in the Yelp2018 dataset, while 52,643 users and 91,599 items as well as 2,984,108 interactions constitute the Amazon-book dataset. The data statistics of the two datasets after processing are provided in Table 3.

Following previous work [2,11], we adopt Recall@K and NDCG@K to evaluate the recommendation performance. Recall@K measures whether the target items are contained in the top-K positions in the recommendation list, while NDCG@K takes the ranking of the target items into consideration, i.e., whether the recommender ranks the target items at right positions. By default, K is set to 20 in our experiments.

### 4.3. Model Summary

To demonstrate the effectiveness of EGCF, we compare our proposed EGCF with the following baselines: (1) Two MF-based methods, i.e., MF [8] and GRMF [14]; (2) A VAEbased model Multi-VAE [32]; (3) Four GNN-based methods, i.e., GC-MC [33], NGCF [2], DGCF [21] and LightGCN [11].

- **MF** [8] utilizes the matrix factorization to exploit the user–item interactions and the BPR loss to optimize the model parameters, where users and items are simply represented by their corresponding IDs.
- **GRMF** [14] introduces the graph Laplacian regularizer to smooth the matrix factorization, where the objective function is changed into BPR loss for fair comparison following [11].
- **Multi-VAE** [32] is an item-based CF method relying on the variational autoencoder (VAE). Here it is assumed that the data is generated from the multinomial distribution and the parameters are estimated by the variational inference.
- **GC-MC** [33] learns the representation of users and items using the GCN encoder, where merely one-hop neighbors are taken into consideration and the hidden size is set as the embedding dimension as in [33].
- **NGCF** [2] models the collaborative signal in the user–item interactions by exploiting the high-order connectivity between users and items using multi-layer GCNs.
- **DGCF** [21] introduces the disentangled learning into graph collaborative filtering to consider user’s diverse interests, which proposes the intent-aware interaction graph to model the distribution over multi intents for each user–item interaction.
- **LightGCN** [11] simplifies NGCF by removing the feature transformation and nonlinear functions in GCN and preserving the most essential component, i.e., neighborhood aggregation for collaborative filtering.

### 4.4. Experimental Setup

Following [2,11,21], we set the embedding size and the batch size to 64 and 1024, respectively, and the Xavier method [34] is adopted to initialize the embedding parameters. We adopt ADAM [35] as the optimizer, where the learning rate is searched in $\left\{1e^{-5},1e^{-4},1e^{-3},1e^{-2}\right\}$ and then set to $1e^{-4}$ for both datasets. Moreover, the L2 regularization is applied for preventing overfitting, which is tuned in $\left\{1e^{-6},1e^{-5},\ldots ,1e^{-2}\right\}$, where the optimal value is $1e^{-4}$ in most cases. In addition, for the trade-off parameters α and λ, we apply a grid search by tuning α and λ in {0.005,0.01,0.05,0.1,0.5} and {6,8,10,12,14}, respectively. Detailed analysis on the trade-off parameters are provided in Section 5.4.

## 5. Results and Discussion

### 5.1. Overall Performance

We present the performance of EGCF and the baselines in Table 4. First, comparing GRMF to MF, we can observe that GRMF outperforms MF obviously in terms of Recall@20 and NDCG@20 on both two datasets, indicating the effectiveness of the graph Laplacian regularizer on smoothing the matrix factorization. Moreover, we can see that the VAE-based method, i.e., Multi-VAE, can achieve better performance than both two MF-based models, i.e., MF and GRMF. This indicates the utility of considering self-supervisions in collaborative filtering since the VAE can be regarded as a special case of the generative self-supervised learning [28].

For the GNN-based methods, first we can observe that GC-MC can outperform MF, indicating the utility of modeling the collaborative signal in the user–item interactions. Moreover, comparing NGCF to GC-MC, it is observed that by exploiting high-order connectivity between users and items, NGCF performs better than GC-MC in terms of both Recall@20 and NDCG@20 on two datasets. In addition, by considering the multi intents of users using the disentangled learning, DGCF further improves the recommendation accuracy and shows obvious superiority than NGCF. Furthermore, by simplifying GCN to improve its appropriateness for recommendation, LightGCN achieves the best performance for all cases on both Yelp2018 and Amazon-book. Thus, we take LightGCN as the baseline for further comparison in the later experiments.

Next, we zoom in on the performance of our proposed EGCF. It is observed that EGCF achieves the state-of-the-art performance for all cases on both Yelp2018 and Amazon-book. We attribute this to that by introducing constrastive learning into the graph collaborative filtering, EGCF can provide sufficient supervision signals to enhance the representation learning of users and items and exploit high-order user–item connectivity without introducing much bias. In addition, the improvement of EGCF above the best baselines, i.e., LightGCN, in terms of Recall@20 and NDCG@20 are 6.40% and 6.86% on the Yelp2018 dataset, respectively, while the corresponding improvements are 11.68% and 13.02% on Amazon-book. First, we can observe that the achieved improvements on both metrics are higher on the Amazon-book dataset than that on Yelp2018. This may be caused by the different scale of two datasets, which indicates that our proposed EGCF can contribute relatively more to improving the performance on larger scale datasets. Moreover, comparing the improvements in terms of Recall@20 and NDCG@20 on two datasets, we can see that the improvement rate is larger on NDCG@20 than that on Recall@20 on both datasets. This indicates that comparing to hitting the target items in the recommendation list, our proposal is more effective on ranking the target items at right positions.

### 5.2. Training Efficiency

**Figure 2:**
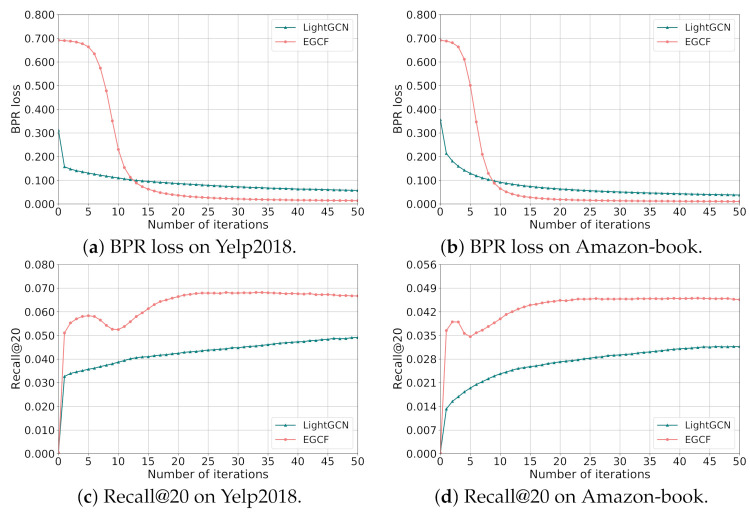
Training curves of LighGCN and EGCF.

For RQ2, in order to investigate the efficiency of EGCF empirically, we measure the training time of EGCF as well as the baseline LightGCN on a single NVIDIA Tesla P100 GPU using both the Tensorflow version code for fair comparison. Specifically, we provide the average training time of each iteration, the number of iterations for the model to converge, and the total training time of LightGCN and EGCF on Yelp2018 and Amazon-book in Table 5. Moreover, we also plot the training curves of the training BPR loss and testing Recall@20 of LightGCN and EGCF until 50 iterations on Yelp2018 and Amazon-book in Figure 2, similar phenomenons to the Recall@20 metric can also be observed in terms of NDCG@20.

From Table 5, we can observe that by avoiding introducing multi-hop neighbors for information propagation in the representation learning of users and items, the training time for each iteration of EGCF is obviously less than LightGCN on both Yelp2018 and Amazon-book. Moreover, the number of iterations for the model to converge decreases by a large margin on both datasets, where EGCF achieves the best performance at the 33-th and 26-th epoch on Yelp2018 and Amazon-book, respectively, while the corresponding iteration numbers for LightGCN are 720 and 700, which are much larger than that for EGCF. This could be explained by the fact that compared with merely adopting the BPR loss for model optimization, the incorporated self-supervisions in constrastive learning can provide sufficient supervision signals, so as to accelerate the model convergence. In addition, we can observe that the decreasing rate of the total training time is 97.91% and 98.47% on Yelp2018 and Amazon-book, respectively, where the acceleration for training is more obvious on Amazon-book than that on Yelp2018. This indicates that EGCF promotes the training efficiency relatively more obviously on large scale datasets.

Moreover, from Figure 2, we can observe that for LightGCN, with the iteration number increasing, the BPR loss keeps decreasing and the performance in terms of Recall@20 keeps increasing on both Yelp2018 and Amazon-book. As for EGCF, we can see that though the BPR loss is higher than that of LightGCN at the first epochs, the BPR loss decreases rapidly from iteration 5 to 12 on Yelp2018 while from iteration 3 to 10 on Amazon-book, and finally achieves obviously lower BPR loss than LightGCN on both datasets. Moreover, we can find an interesting phenomenon that while the BPR loss of EGCF keeps decreasing, the performance in terms of Recall@20 on two datasets both fluctuates during training. We analyze the possible reason is that the supervised learning focuses on ranking user’s interacted items earlier than other items, while constrastive learning aims to pull similar users or items together and push dissimilar users or items apart in the embedding space, which may lead to the instability of training to some extent when combining them together. However, by choosing a proper α for joint learning, EGCF can finally achieve obviously better performance than LightGCN.

### 5.3. Ablation Study

For RQ3, to prove the effectiveness of each component in EGCF, we perform an ablation study by comparing EGCF with its variants, which includes:

- **w/o GNN** removes the neighbor information obtained by Equation (1) from Equation (2) for the representation learning of users and items.
- **w/o CL** removes the contrastive loss obtained by Equation (7) from EGCF.
- **w/o I-CL** removes the item-side contrastive loss obtained by Equation (5) from EGCF by removing $L_{c}^{i}$ from Equation (7).
- **w/o U-CL** removes the user-side contrastive loss obtained by Equation (6) from EGCF by removing $L_{c}^{u}$ from Equation (7).

**Figure 3:**
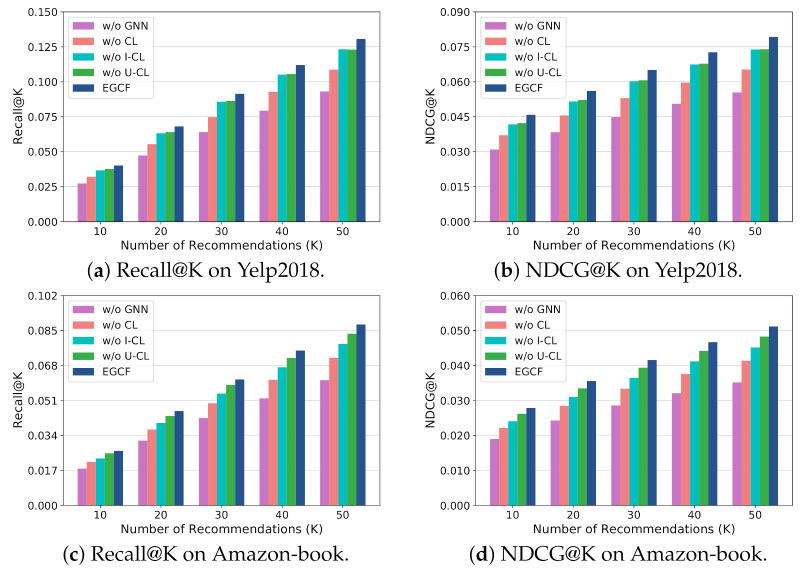
Ablation study.

The results of EGCF as well as the variants are presented in Figure 3, where we evaluate all models by ranging the recommendation number, i.e., K, in {10,20,30,40,50} to provide a comprehensive comparison.

From Figure 3, we can observe that removing each component from EGCF will cause the performance dropping obviously in terms of both Recall@K and NDCG@K with various K on two datasets, indicating the utility of each component on promoting the recommendation accuracy of EGCF. Moreover, increasing the number of recommended items will consistently increase the performance in terms of both Recall@K and NDCG@K, since long recommendation lists are more likely to include the target items. Moreover, compared with w/o CL, w/o GNN which further removes the neighbor information for representing users and items, generally underperforms w/o CL for all cases on two datasets, indicating the utility of modeling the collaborative signal in the user–item interactions for recommendation.

In addition, comparing w/o I-CL and w/o U-CL, we can find that on Yelp2018, w/o I-CL and w/o U-CL have comparable performance in terms of both Recall@K and NDCG@K with K ranging from 10 to 50. However, differently on Amazon-book, w/o U-CL generally achieves better performance than w/o I-CL. We attribute this phenomenon to the different number of users and items on two datasets. More specifically, item-side contrastive learning can introduce self-supervisions for distinguishing the items while the user-side one can help distinguish users as explained in Section 3.2.2. Thus, similar number of items and users leads to the similar impact of item- and user-side contrastive learning on the performance on Yelp2018. However, on Amazon-book, a larger number of items than users causes a more obvious impact of the item-side contrastive learning on the performance, which explains the larger performance dropping when removing the item-side contrastive learning.

### 5.4. Hyper-Parameter Analysis

In order to investigate the impact of the hyper-parameters α and λ on the performance of EGCF, we apply a grid search which tunes α and λ in {0.005,0.01,0.05,0.1,0.5} and {6,8,10,12,14}, respectively. We provide the results on Yelp2018 in Table 6, while the results on Amazon-book are shown in Table 7.

First, we can observe that EGCF achieves the best performance in terms of Recall@20 and NDCG@20 on both datasets at α = 0.05 and λ = 8. For the Yelp2018 dataset, from Table 6, we can see that for each α, with λ increasing, the performance of EGCF in terms of both Recall@20 and NDCG@20 first increases and then decreases. Moreover, for each λ, with α increasing, the performance of EGCF in terms of both metrics also shows a similar trend. We analyze the possible reason is that the intensity of introducing self-supervisions with different hyper-parameters is various. Specifically, a large α can increase the impact of contrastive learning without doubt, while for the parameter λ, a large λ indicates that the users and items are distinguished more obviously in the InfoNCE as indicated in multi work [13,36], which also increases the intensity of contrastive learning. This explains the above phenomenons since small α and λ cannot provide sufficiently enough self-supervisions, while large α and λ may lead the model to overfitting. Similar phenomenons can also be observed on the Amazon-book dataset from Table 7.

#### 5.4.1. Impact of Parameter α

**Figure 4:**
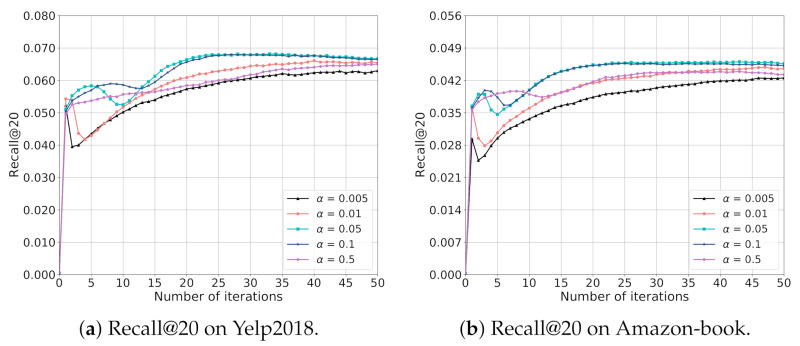
Impact of α on the model performance.

In order to provide more detailed analysis on the impact of α on the model performance, we evaluate the performance of EGCF by tuning α in {0.005,0.01,0.05,0.1,0.5} and fixing λ as 8, the training curves in terms of Recall@20 on two datasets are provided in Figure 4, similar phenomenons can also be observed on the NDCG@20 metric.

From Figure 4, we can observe that the peak performance of EGCF with α = 0.005 and α = 0.5 is obviously lower than EGCF with other α, which is due to that they fail to provide enough self-supervisions and introduce redundant contrastive signals, respectively, limiting the model performance. Moreover, we can see that most curves fluctuate at the first epochs, except EGCF with α = 0.5 on Yelp2018 which keeps increasing when the iteration number increases. In addition, with α decreasing, the fluctuation of the performance curve becomes more obvious, indicating that small α is easier to cause the instability of training.

#### 5.4.2. Impact of Parameter λ

**Figure 5:**
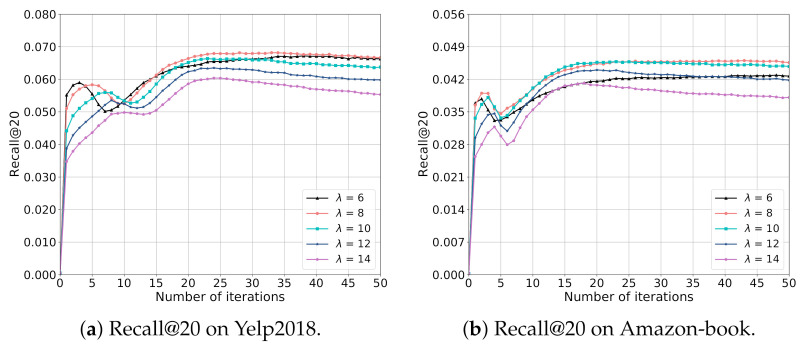
Impact of λ on the model performance.

To analyze the impact of λ on the recommendation accuracy in more detail, we provide the training curves of EGCF in terms of Recall@20 on both Yelp2018 and Amazon-book with ranging λ in {6,8,10,12,14} and fixing α as 0.05 in Figure 5, similar phenomenons can also be observed in terms of NDCG@20.

From Figure 5, we can see that EGCF with λ = 8 can achieve the best performance in terms of Recall@20 on two datasets, where increasing or decreasing λ will both cause the performance dropping. This could be explained by that λ in the InfoNCE influences the distribution of users and items in the embedding space [13], where increasing λ will lead the model to learning more discriminative embeddings of users and items. However, large λ may cause the overfitting problem and small λ cannot distinguish different users and items effectively, which both decrease the model performance.

## 6. Conclusions and Future Work

In this paper, we propose a novel approach—Efficient Graph Collaborative Filtering (EGCF). First, EGCF applies merely one-layer graph convolution to generate the representation of users and items from the user–item interactions for taking the collaborative signal into consideration. Moreover, we introduce constrastive learning to derive the self-supervisions for enhancing the representation learning of users and items and exploiting the high-order connectivity between users and items. Comprehensive experiments conducted on two benchmark datasets validate the effectiveness of EGCF in terms of Recall and NDCG, especially on ranking the target items at right positions. Moreover, the high training efficiency of EGCF makes it practicable for real-world potential applications.

As to future work, we would like to further improve the efficiency of EGCF, such as adopting the non-sampling strategies [37,38] for model optimization. Moreover, we are also interested in incorporating the side information such as knowledge graph [39,40] to alleviate the sparsity and cold start problems in collaborative filtering.

## Figures and Tables

**Table 1:** Main notations used in this paper.

Notation	Description
U	the user set containing all users
V	the item set containing all items
$O^{+}$	the observed interactions between U and V
*d*	the dimension of the user and item embeddings
G={V,E}	the user–item bipartite graph constructed from $O^{+}$
E	the initial item embeddings of nodes in V
Z	the item representations of nodes in V learnt by SGCN
$N_{u},N_{i}$	the neighbors of user *u* and item *i* in the bipartite graph
$a_{u},a_{i}$	the propagated information for user *u* and item *i*
${\hat{y}}_{ui}$	the prediction score of user *u* on item *i*
$L_{s}$	the supervised Bayesian personalized ranking loss
λ	the trade-off parameter for scaling the cosine similarity
$L_{c}^{i},L_{c}^{u},L_{c}$	the item-side, user-side and combined contrastive loss
α	the trade-off parameter for balancing $L_{s}$ and $L_{c}$
L	the combined loss for joint learning
*L*	the layer number of GNNs
*s*	the number of training epochs for model optimization
*B*	the size of each training mini-batch
K	the number of items recommended to the user

**Table 2:** Comparison of computational complexity between LightGCN and EGCF.

Component	LightGCN	EGCF
Adjacency Matrix	O(2|E|)	O(2|E|)
Graph Convolution	$O(2|E|Lds\frac{\left|E\right|}{B})$	$O(2|E|ds\frac{\left|E\right|}{B})$
Supervised Loss	O(2|E|ds)	O(2|E|ds)
Contrastive Loss	-	O((2+2B)|E|ds)

**Table 3:** Dataset statistics.

Dataset	#Users	#Items	#Interactions	#Density
Yelp2018	31,668	38,048	1,561,406	0.00130
Amazon-book	52,643	91,599	2,984,108	0.00062

**Table 4:** Model performance. The results of the best performing baseline and the best performer in each column are underlined and boldfaced, respectively. ${}^{▴}$ denotes a significant improvement of EGCF over the best baseline using a paired *t*-test (*p* < 0.01).

Method	Yelp2018		Amazon-Book	
	Recall@20	NDCG@20	Recall@20	NDCG@20
MF	0.0433	0.0354	0.0250	0.0196
GRMF	0.0571	0.0462	0.0354	0.0270
Mult-VAE	0.0584	0.0450	0.0407	0.0315
GC-MC	0.0462	0.0379	0.0288	0.0224
NGCF	0.0579	0.0477	0.0344	0.0263
DGCF	0.0640	0.0522	0.0399	0.0308
LightGCN	0.0641	0.0525	0.0411	0.0315
**EGCF**	**0.0682** ${}^{▴}$	**0.0561** ${}^{▴}$	**0.0459** ${}^{▴}$	**0.0356** ${}^{▴}$

**Table 5:** Comparison of runtime between LightGCN and EGCF. Here, “S”, “I” and “T” indicate the average training time for each iteration, the iteration number for the model to converge and the total time for training, respectively, while “s” and “m” denote “second” and “minute”, respectively.

Method	Yelp2018			Amazon-Book		
	S	I	T	S	I	T
LightGCN	22.19 s	720	266.28 m	85.07 s	700	992.48 m
EGCF	10.11 s	633	665.56 m	34.97 s	626	615.15 m

**Table 6:** Impact of the hyper-parameters α and λ for Yelp2018.

Recall@20					
	λ=6	λ=8	λ=10	λ=12	λ=14
α = 0.005	0.0512	0.0524	0.0524	0.0643	0.0637
α = 0.01	0.0536	0.0543	0.0549	0.0652	0.0637
α = 0.05	0.0590	**0.0682**	0.0664	0.0635	0.0604
α = 0.1	0.0679	0.0680	0.0652	0.0614	0.0576
α = 0.5	0.0665	0.0650	0.0603	0.0554	0.0507
NDCG@20					
α = 0.005	0.0413	0.0428	0.0429	0.0526	0.0523
α = 0.01	0.0440	0.0445	0.0451	0.0535	0.0524
α = 0.05	0.0484	**0.0561**	0.0545	0.0523	0.0497
α = 0.1	0.0552	0.0559	0.0539	0.0508	0.0474
α = 0.5	0.0544	0.0534	0.0496	0.0458	0.0420

**Table 7:** Impact of the hyper-parameters α and λ for Amazon-book.

Recall@20					
	λ=6	λ=8	λ=10	λ=12	λ=14
α = 0.005	0.0420	0.0436	0.0447	0.0446	0.0432
α = 0.01	0.0424	0.0450	0.0460	0.0451	0.0433
α = 0.05	0.0429	**0.0459**	0.0458	0.0440	0.0412
α = 0.1	0.0428	0.0457	0.0454	0.0429	0.0399
α = 0.5	0.0391	0.0439	0.0425	0.0391	0.0350
NDCG@20					
α = 0.005	0.0324	0.0336	0.0347	0.0344	0.0335
α = 0.01	0.0327	0.0348	0.0356	0.0349	0.0337
α = 0.05	0.0332	**0.0357**	0.0356	0.0342	0.0320
α = 0.1	0.0332	0.0353	0.0352	0.0334	0.0310
α = 0.5	0.0310	0.0337	0.0328	0.0304	0.0274

## Data Availability

The datasets can be found at https://www.yelp.com/dataset/challenge and http://jmcauley.ucsd.edu/data/amazon (accessed on 20 May 2021).
